# Consistency and Standardization of Color in Medical Imaging: a Consensus Report

**DOI:** 10.1007/s10278-014-9721-0

**Published:** 2014-07-09

**Authors:** Aldo Badano, Craig Revie, Andrew Casertano, Wei-Chung Cheng, Phil Green, Tom Kimpe, Elizabeth Krupinski, Christye Sisson, Stein Skrøvseth, Darren Treanor, Paul Boynton, David Clunie, Michael J. Flynn, Tatsuo Heki, Stephen Hewitt, Hiroyuki Homma, Andy Masia, Takashi Matsui, Balázs Nagy, Masahiro Nishibori, John Penczek, Thomas Schopf, Yukako Yagi, Hideto Yokoi

**Affiliations:** 1 Division of Imaging and Applied Mathematics, Center for Devices and Radiological Health, U.S. Food and Drug Administration, 10993 New Hampshire Ave., 20993 Silver Spring, USA; 2 FFEI Limited & International Color Consortium, Hemel Hempstead, London, UK; 3 S.A.N. Business Consultants, LLP, New York City, USA; 4 Gjovik University College, Tromsø, Norway; 5 Barco NV, Healthcare Division, Kortrijk, Brussels, Belgium; 6 Department of Medical Imaging and Arizona Telemedicine Program, University of Arizona, Tucson, AZ USA; 7 School of Photographic Arts & Sciences, Rochester Institute of Technology, Rochester, NY USA; 8 Norwegian Center for Integrated Care and Telemedicine, University Hospital of North Norway, Tromsø, Norway; 9 Leeds Teaching Hospitals NHS Trust and University of Leeds, London, UK; 10 National Institute of Standards and Technology, Gaithersburg, USA; 11 PixelMed Publishing, Philadelphia, PA USA; 12 Henry Ford Health System, New York City, USA; 13 Healthcare Business Development Office, Fujifilm Corporation, Tokyo, Japan; 14 Center for Cancer Research, National Cancer Institute, Bethesda, USA; 15 Olympus Medical Systems Corporation, Tokyo, Japan; 16 X-Rite, Boston, USA; 17 Eizo Corporation, Tokyo, Japan; 18 Department of Experimental Psychology, Institute of Psychology, Sao Paulo, Brazil; 19 Department of Social Services and Healthcare Management, International University of Health and Welfare, Tokyo, Japan; 20 National Institute of Standards and Technology and the University of Colorado, Boulder, CO USA; 21 Norwegian Center for Integrated Care and Telemedicine, University Hospital of North Norway, Tromsø, Norway; 22 Department of Medical Informatics, Kagawa University Hospital, Tokyo, Japan; 23 Harvard Medical School and Massachusetts General Hospital, Boston, MA USA; 24 Center for Neuroscience and Behavior and Department of Mechatronics, Optics and Engineering Informatics, Budapest University of Technology and Economics, Budapest, Hungary

**Keywords:** Color imaging, Medical imaging, Color calibration, Color management

## Abstract

This article summarizes the consensus reached at the Summit on Color in Medical Imaging held at the Food and Drug Administration (FDA) on May 8–9, 2013, co-sponsored by the FDA and ICC (International Color Consortium). The purpose of the meeting was to gather information on how color is currently handled by medical imaging systems to identify areas where there is a need for improvement, to define objective requirements, and to facilitate consensus development of best practices. Participants were asked to identify areas of concern and unmet needs. This summary documents the topics that were discussed at the meeting and recommendations that were made by the participants. Key areas identified where improvements in color would provide immediate tangible benefits were those of digital microscopy, telemedicine, medical photography (particularly ophthalmic and dental photography), and display calibration. Work in these and other related areas has been started within several professional groups, including the creation of the ICC Medical Imaging Working Group.

## Background

Today, color in medical imaging specialties is handled, with few exceptions, in an ad hoc manner with little standardization. In some areas, this presents a number of challenges to medical professionals who wish to use color images for diagnostic purposes.

To understand color properties of medical imaging systems, one can refer to the different stages of the imaging process often referred to as the imaging chain. In all imaging systems, there exist a source of radiation, an object to be imaged, an image capture device, and a processing stage that can include image analysis and manipulations, and image storage. The final stage in most imaging systems where the images are interpreted by humans is the display device and visualization approaches. Each step in this chain can be characterized by the properties of each component that ultimately determine image quality, in this case, with respect to the handling of information encoded in color. In this context, color standardization and consistency can only be achieved if at all stages of the imaging chain, the system designer recognizes the effects of each component on the transfer of color information. In this paper, we review the different areas where standardization is required to improve the consistent handling in medical images for a variety of color-critical imaging applications.

In general, color image capture, processing, and storage and display are all affected by a variety of hardware and software components, often from different manufacturers. In order to achieve consistency and interoperability, every component of the system must have a clear description of what is expected for color presentation. For example, images from different modalities may be processed using a variety of image processing software and viewed on different types of display devices using viewing software from different manufacturers. Given this diversity, it is important for all those involved to adopt a clearly defined color architecture that supports all types of color images and has a high level of interoperability across devices.

In the near future, the integrated patient record and electronic health record will provide all data related to a patient including images, providing a complete record of medical procedures. Given the current rate of development of health care information technology infrastructure, these data will be easily accessible with image data from all modalities, and they are likely to be presented on the same display to provide a single point of access. To achieve a high level of image fidelity, a well-defined color framework for transfer of color information is required.

Within that framework, there are two distinct aspects to consider: color accuracy and color consistency. Color accuracy refers to the ability of the system to produce exact color matches from input to output and it is typically measured for a set of colors. On the other hand, color consistency is a vaguer term that refers to the ability of the system or systems to produce image data with an identical (or similar) perceptual response in the human interpreter. While accuracy can be defined for a single system since it refers to the relationship between input and output data, we can formulate intra- and intersystem consistency metrics, In some cases, maintaining color accuracy is important, that is, colors should remain unchanged (within system tolerances) from capture to display. There are, however, many cases where color image processing can highlight image features to show some aspect of the data more clearly and in such cases consistency in the way in which color is presented is important. Overall, the requirements for color reproducibility and standardization depend on the intended use of the images since human observers can be more tolerant of variability than image analysis algorithms. Both aspects of accuracy and consistency require standard methods and test objects that provide the means of characterizing the system performance in a well-defined manner.

## Current Practices and Challenges

The following sections represent descriptions of current challenges in the use of color for a variety of medical disciplines that rely on color images for diagnostic purposes. Within each section, we summarize the main consensus recommendations for addressing the issues and challenges described. Color applications in medical imaging include color for annotations and thresholding where color is not usually critical, pseudo-color applications where the displayed color has no correlation with the color of the object being imaged (e.g., visualization of calculated data and fused images), and, finally, true-color applications where the displayed color is intended to represent the actual color of the imaged object (e.g., dermatology and medical photography).

## Whole-Slide Imaging, Digital Microscopy, and Histopathology

**Fig. 1:**
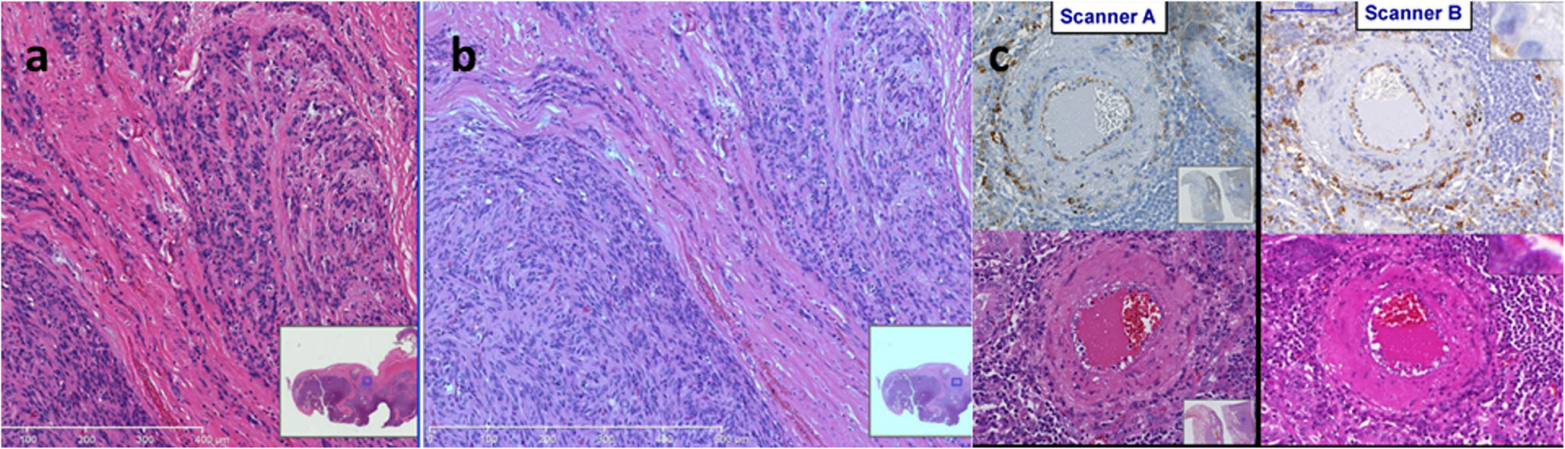
Observed variation in color between scanners and software. **a**, **b** The same slide imaged with the same scanner, viewed using two different software packages (screenshots). **c** The same slide imaged on two different scanners with IHC (*top*) and H&E (*bottom*) stains. The color rendition of the scans appears noticeably different from scanner A to B

Although a variety of modalities in these disciplines utilize digital images including telepathology and digital microscopy, whole-slide imaging (WSI) is the modality most likely to drive the adoption of color standardization. Applications include research and clinical uses of pathology imaging for visual assessment, and (importantly) the automated assessment of images with image analysis software algorithms. Pathology samples are stained with a wide variety of colored dyes. With conventional light microscopy and human interpretation, variability in color is often not a critical issue, perhaps because in such a viewing environment, it is easy to adapt to differences and it is often easy to simply stain another piece of the tissue sample. The increasing use of digital pathology, however, highlights the color differences between samples, laboratories, and scanning and display systems (see Fig. 1). There are noticeable color differences in these situations, which are visible to the observer and affect the results of image analysis algorithms.

There is a significant variability in the color of images in this domain introduced at almost all stages of the process from sample acquisition through slide preparation, imaging, and transmission to the display device. A distinction can be drawn between color variation occurring before imaging (a significant task requiring standardization of tissue acquisition, fixation, processing, and staining) and during or after imaging (addressable using end-to-end color calibration techniques).

Initial efforts to standardize color using various color transformations have been described [1]. However, color accuracy is only one factor affecting the “fitness for purpose” of WSI for diagnostic use. Other factors include the resolution, contrast, and dynamic range of the image. Because there is significant variability in histochemical staining between and within laboratories, standardizing all pre-imaging sample handling steps (acquisition, fixation, processing, sectioning, and staining—known as “pre-analytic variables”) is a difficult task. Even with these additional variables, imaging systems should be able to generate an image with consistent color presentation irrespective of the instrumentation or devices used.

In addressing the variability introduced during and after imaging, current major roadblocks include the significant variation in performance between WSI scanners (even those from the same vendor), the lack of standardized color calibration, and the difficulty in designing and constructing appropriate test objects for scanners. Preliminary work in developing such a target has been conducted [1]. Key challenges include obtaining agreement between pathologists on what is the ideal color for a stain, defining end-to-end image standardization standards, and developing technology and infrastructure to apply standardization.

For WSI, end-to-end color management within the digital pathology workflow is required with special emphasis to the imaging chain stages of image capture, processing, and display. Ideally, this can be achieved with a multidisciplinary approach involving pathologists, color scientists, engineers, and digital pathology vendors with appropriate “targets” for scanner calibration. Previous work has showed that the application of appropriate color mappings can reduce interimage variability. In addition, there is no standard method of assessing dynamic range and there is some subjective evidence that the current implementations of WSI systems may not retain sufficient dynamic range from capture to display. The choice of the illumination condenser and light source plays a role in image quality. Although stains applied to tissue obey Beer’s law, where absorbance is proportional to concentration, some histologic preparations are impregnations or result in the deposition of particles that result in the refraction of light, rather than absorbance. As a result, silver-impregnations, and particle-based detection of IHC (DAB chromogen) are visualized based on their refractive properties. Depending on the optical path geometry and illumination design of the instrument, these objects may not replicate what is observed in manual microscopy.

Consistent color approaches in WSI require efforts to characterize and address the problem of color variation, including collaborative work on developing a test object. An intermediate step is to formally investigate the extent of color variation between scanners, and reasons for this variability. For example, appropriate test or calibration slides may reveal differences between scanner optics, on-board software or image storage and transmission. Such an effort will require cooperation from WSI users and vendors. Long-term aims for this work should include international standards for color reproduction, technology to apply standardization from the glass slide to the display, and a better understanding in the general pathology community of the importance of such work.

Furthermore, agreement on standardized tissue handling protocols and stain standardization may be useful at the preimaging stage including input from regulatory bodies such as the US Biological Stains Commission and equivalent international organizations. Further study of the causes of variation in color at the preimaging stage may also be necessary in the broader context of reducing variability in pathology images and automatic and computer quantitative analysis of images.

## Endoscopy and Laparoscopy

Endoscopes are used by almost every medical specialty: gastroenterology, pulmonology, gynecology, urology, orthopedics, anesthesia, ENT, thoracic surgery, and general surgery. Color plays an important role in endoscopic [2] as well as in laparoscopic [3] imaging. The main purpose of endoscopy/laparoscopy is to visualize a certain object inside the human body, such as internal organs, from outside. To visualize the internal organs, one must deliver the light into the human body to shine the object, image the scene, transmit the image data back to the outside, and finally reproduce the image for human readers. Normally, internal organs in vivo are not visible by a naked eye because the human body blocks visible light. Thus, the key of various endoscopy and laparoscopy technologies is to get around the barrier such that the light can be delivered and the image can be retrieved. Different devices address this problem with different approaches: the straightforward method is to perform surgery (e.g., thoracic or general) and gain direct access to the object. Laparoscopy is a less intrusive alternative to surgery. Endoscopy gains access through the cavities of the human body.

Conventionally, a tubular device is used to house both the light delivery channel and the image retrieval channel for either passing through or getting around the barrier. Capsule endoscopy takes advantage of the open circuit of the human gastrointestinal tract. The capsule travels the gastrointestinal tract passively while capturing and storing the images autonomously. Table 1 compares various endoscopic and laparoscopic methods by parameterizing each component in the imaging chain with examples.

**Table 1:** Endoscopic/laparoscopic methods with imaging components and examples

Object	Illumination	Light guide	Imaging mode	Image detector	Image guide	Intermediate image/video file	Video processor	Display
Open surgery	Lighting in operation room	None	Surgeon	Human visual system	None	None	None	None
Endoscopy and laparoscopic surgery	Proximal: xenon lamp, halogen lamp, filtered narrow band	Optical fiber	Rigid and flexible direct viewing scope	Human visual system	Optical lens/fiber optic bundle	None	None	None
			Rigid and flexible video scope	Distal sensor	Electrical wired	Optional	Real-time	On-site PACS EMR
	Distal: LED	None						
			Capsule	Distal sensor	Wireless/radio transmission or internal storage	Yes	Off-line compilation	

A critical aspect for identifying color-related issues in endoscopic and laparoscopic systems is a review of the makeup of the imaging chain. The generalized imaging chain consists of object, illumination, light guide, detector, image guide, intermediate image or video file, video processor, display, and human reader. In laparoscopy systems, the illumination source is typically a xenon lamp. In endoscopy, the illumination source can be xenon/halogen light, filtered narrow-band light guided by a fiber optic bundle at the proximal end, or light-emitting diode lights embedded at the distal end. The endoscope itself can be rigid, flexible, or in a capsule form. The image detector can be an external video camera at the proximal end, or an embedded camera at the distal end. While most flexible endoscopes transmit image data electrically via internal wires, capsule endoscopes can transmit image data electrically via human body, electromagnetically via radiofrequency signaling, or physically via memory storage. The capsule systems also require the image data to be saved as intermediate files for off-line processing and review. The video data is rendered by the video processor in real time for flexible endoscopes, or off line for capsule endoscopes. The video data is presented to the endoscopist by an electronic display device during the procedure and can be archived in a picture archiving and communications systems (PACS) or as part of the electronic medical record (EMR) system for future review.

In these modalities, it is important to clarify related topics: color reproducibility, color consistency, color characterization, and color standardization. Color reproducibility means that the original scene can be faithfully reproduced, either colorimetrically or perceptually, on the final display. Color consistency means that the relationship between the input scene and the output image (i.e., the color mapping) remains constant. Color characterization means to measure and to determine the relationship between the input and output of a component/system with a certain color target. Color standardization means that the input and output of a component in the imaging chain are well defined such that products from different vendors are interchangeable (i.e., interoperability).

Surgeons who perform both open surgery and laparoscopic surgery may see slight differences in color when observing the same tissue. During open surgery, the surgeon looks at the tissue directly with his/her naked eyes while the tissue is illuminated by the lighting in the operating room. During laparoscopic surgery, the same surgeon observes the same tissue via the laparoscope and can therefore compare the color of the laparoscopic image to his/her recollection of how the tissue appeared when viewed directly with the naked eye. However, in a video-based (e.g., flexible or capsule) endoscopic procedure, the endoscopist observes a reproduced electronic image of the object illuminated by a nonstandard light source. If the endoscopist does not normally see this same tissue with his/her naked eye, but always uses an endoscope, the endoscopist believes the true color of the tissue is similar to the endoscopic image. Results from surveys of endoscopists and laparoscopists described at the Summit demonstrate that although a true color match between real-life color and the color on the display appears to be more important for laparoscopy than for endoscopy, most participants agreed that color standardization is beneficial for the advancement of the technology and its clinical applications. However, no misdiagnoses have been reported due to unfaithful color reproduction. Endoscopists rely on a pathologist’s examination of the endoscopic biopsy for the diagnosis. Therefore, nonideal color reproducibility of endoscopy/laparoscopy devices does not lead to safety concerns.

Achieving color reproducibility remains difficult since current endoscopes cannot emit light evenly yielding color variations with brightness. To date, the design goal of current endoscopy systems is not color reproducibility but user preference. In addition, since determining the original scene with existing endoscope products is not feasible, one can use the image shown by the onsite display to the endoscopist for establishing the diagnosis truth. However, color characterizing the onsite display is also challenging (see the Medical Display Section) and providing an identical or similar image on other display devices remains difficult even with a modification of the digital imaging and communications in medicine (DICOM) standard. This level of color consistency for the display of color images requires characterizing the input and output devices and adding new tags into the DICOM or ICC format, which are feasible but practically challenging. Current devices include quality assurance/quality control (QA/QC) procedures and criteria not based on the original scene but on the color consistency requirements imposed by the manufacturers.

In summary, color standardization is not critical in endoscopy if the components of the system are from the same vendor and managed within a unique framework. When systems consist of varying components or devices from different vendors, standardization should be helpful for achieving consistency. Even though the laparoscopic system is also designed in the same manner, it is physically possible to be composed of mix-and-match components from different vendors and thus color standardization would be preferred. In addition, color standardization is important if color consistency is required when endoscopy video is archived or recorded for future review with identical or completely different systems. Overall, faithful color reproduction is technically challenging for these modalities and currently not in demand by the user community.

## Ophthalmology (Fundus and Retinal Imaging)

**Fig. 2:**
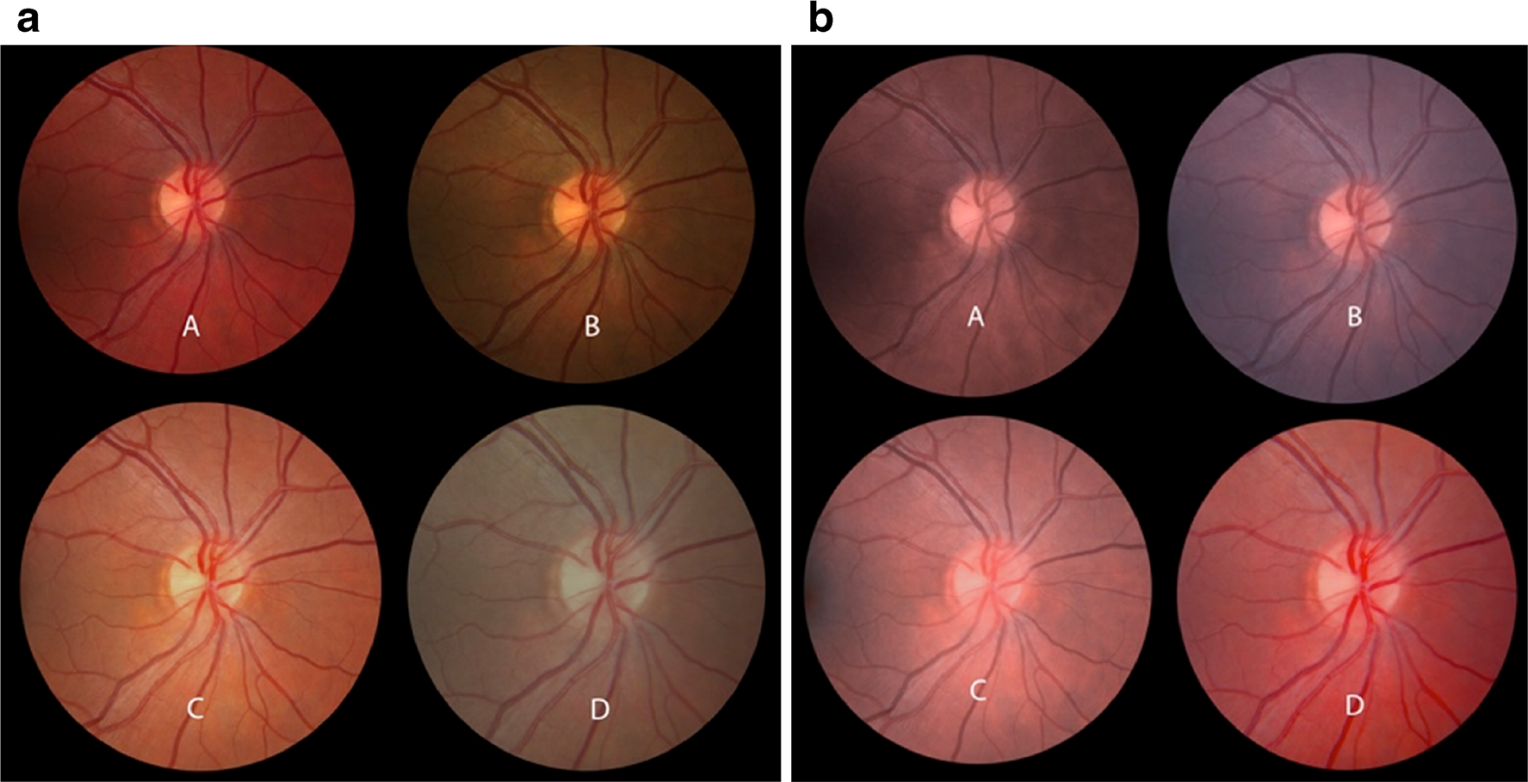
*Left panel* observed variability in the color obtained by imaging the same patient on four different fundus cameras from three different manufacturers. *Right panel* fundus images obtained by applying color camera profiles after capture

Mydriatic fundus photography is one of the most prevalent forms of retinal imaging. This type of imaging is used in a clinical ophthalmology setting, most commonly as a mechanism of documenting the visual appearance of a patient’s retina on the day of their visit. It is also used as a secondary tool in clinical trials and research, as well as in teleophthalmology, as replacement for in-person patient visits. The relevance of color in all of these contexts pertains to accuracy as well as consistency [4, 5]. In order for the physician to be satisfied that the image is a reasonable reproduction of the patient’s retina as a medical record, it should match, as closely as possible, the view the ophthalmologist has during examination with an indirect ophthalmoscope. Consistency of color includes consistency between images from a single camera as well as between different cameras or cameras from different manufacturers [6–9]. Digital fundus cameras can be compared by photographing the same eye with each camera and analyzing the differences between them using a known standard as reference with results showing significant variability from the same patient and same eye (see Fig. 2). Testing can also include photographing several known test color patches inside an eye model with all cameras and averaging the final captured color at the center of the image.

**Fig. 3:**
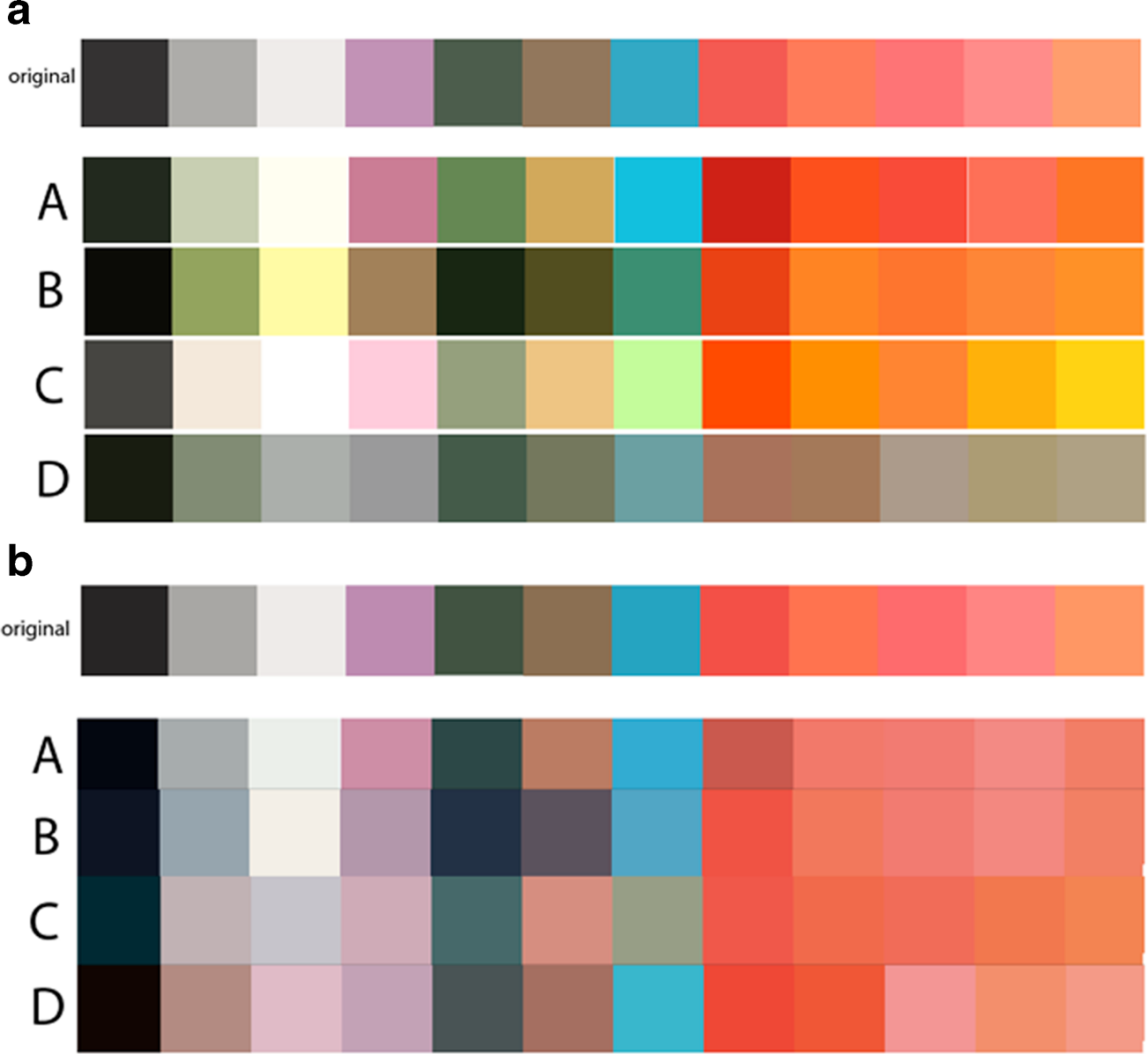
Color patches **a** captured in each fundus camera inside a model eye and **b** image-processed to match original targets

Typically, digital photographic cameras do not allow direct file access. Investigations on what differences may exist between processed (TIFF) and unprocessed (RAW) file formats in terms of color differences will contribute to understand the effect of proprietary processing after capture. The most significant barrier to the implementation of color correction and management in ophthalmology is the lack of standardization. Identification of a standard set of colors and a common method of imaging these colors in order to generate color profiles is needed. A common method inspired on established methodologies based on the Macbeth Color Checker (see Fig. 3 for an example test target proposed for system calibration), with an eye model and a standardized approach for profile generation, would result in more consistent color across cameras and systems.

## Medical Photography and Related Modalities

**Fig. 4:**
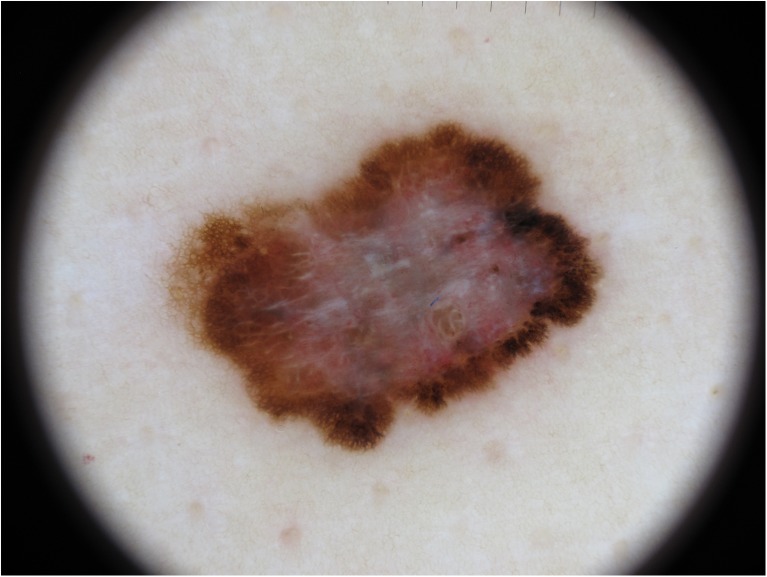
Dermoscopy picture of a malignant melanoma of the skin. The characteristic blue-white veil has high diagnostic value but requires precise color rendering to be identifiable (image courtesy of Dr. Herbert Kirchesch)

Medical photography uses noninvasive visible-light photographs captured by off-the-shelf or specialized equipment. These include whole-body photographs and detailed close-up photography of the skin, including digital dermoscopy (see Fig. 4) and multispectral imaging [10], primarily for diagnostic purposes, requiring precise color rendering to avoid erroneous conclusions. Typical use cases are in telemedicine applications with a remote specialist having access only to the image and supplementary information about the patient or as input to a computer-aided diagnostic tool where color is a feature in a machine learning classification [11]. In these cases, precision and consistency of color reproduction is critical to achieve correct clinical decisions. Inconsistency and poor accuracy in color rendering in the recording, communication, and interpretation stages cannot only affect the telemedicine diagnosis, but also the subsequent evaluation of effectiveness of treatment based on image records. Color accuracy is sensitive to the image capture process and varies widely depending on the equipment, method, and lighting environment. Camera settings, most critically white balance, and ambient light conditions must both be considered in the capture process to obtain optimal color rendering [12]. Most casual users of photographic equipment are not trained in how to employ best practices for acquiring color images, which inevitably leads to unacceptably large variation in color. Greater training and a reliable process are needed to improve the color quality of medical images.

The establishment of best practices guidelines for the photography of color medical images and the definition of a set of reference test targets or images for various modalities can contribute to ensure consistent color renderings. In addition, efforts in this specialty should include the use of standard image post-processing color correction methods. This would allow an investigation into the way diagnostic errors relate to inconsistency of color reproduction in various conditions and modalities.

## Dental Photography

In dentistry, photographs are widely used by a diverse group of stakeholders who need to be able to communicate effectively with each other. Stakeholders include dental providers, technicians, dentists, specialists (including orthodontists), prosthodontics, periodontics, endodontists, oral and maxillofacial radiologists, pathologists and surgeons, dental manufacturers, laboratories, and camera hardware and software vendors. Color fidelity in dental photography is relevant in the clinical areas of treatment planning, esthetic dentistry and implant treatment, and in root fracture risk assessment [13-20]. In diagnosis and treatment planning, providers look for pockets, plaque deposits, and abscesses to treat diastema gingiva alteration, chin remold, and malocclusions alterations, all of which are affected by color techniques.

Prosthodontics use visible light photography for matching shade, fit and restoration of implants, crowns, bridges, veneers, inlays, and the complete and removable partial dentures. When making decisions about color, the clinician must consider the lighting conditions at the image acquisition stage including the color temperature of the light source. In periodontics, studying and treating diseases of the periodontium tissue as well as placement and maintenance of dental implants requires direct, real-time visualization and magnification of the subgingival tooth root surface and recessions, aiding the identification of deposits on the tooth surface.

Color is also of relevance in esthetic dentistry where color matching includes such nuances as hue, tint, translucency, refractive index, and these depth-related textures vary substantially across the visible crowns of individual teeth and need matching with their contralateral front counterparts. These subtle variations need to be recorded and accurately reproduced during preparation of prosthetic tooth crowns. Oral and maxillofacial surgery includes extractions, implants, and facial surgery. Surgeons diagnose and treat oral cancer and other diseases in the maxillofacial region. They use visible light photographs to monitor changes in color, detect white spot lesions, and gray and black discolorations to help with oral cancer assessment. In oral and maxillofacial pathology, both clinical disease progress of soft tissue lesions and also color in light microscopy of biopsy specimens are important. In endodoncy, tooth discoloration is an important determinant of pulp short-term bruising versus necrosis. Endoscopes also are commonly used to look inside the tooth roots during root canal preparation for root apex obituration.

In summary, proper handling of color in dentistry is hindered by lack of interconnectivity and consistency among proprietary systems. These effects can be minimized by providing education on color spaces and their influence on imaging tasks to dental health providers including training on the effect of the spectral characteristics of the ambient illumination through technical reports from recognized industry bodies such as the American Dental Association. In addition, implementing the DICOM WG 22 (Dentistry) color framework with appropriate targets and phantoms for color calibration of dental imaging systems will contribute to achieving consistency while ensuring interconnectivity among systems through integrating the healthcare enterprise (IHE) profiles.

## Display Systems

Display systems are key components for the visualization of color medical images as evidenced by recently published reports. Display devices and approaches are relevant to the discussions of previous section in this article whenever the color image is to be presented to a human. Recent work demonstrated that color displays suffer from limited primary stability [21], which leads to color gamut shrinkage, color shift, gray imbalance, and contrast reduction. A quantitative method was provided to measure primary stability. In addition, color displays have large variability related to color of the white point, color point of neutral gray values, and even color differences within the entire color gamut, even when color management experiments attempt to reproduce different tone curves and white points on the display screen adjusted to DICOM grayscale standard display function (GSDF) [22].

Color calibration of displays for medical applications is necessary to guarantee stability of display systems over time and to ensure similar behavior between different display brands and types. There is no strong clinical evidence that such calibration increases diagnostic efficacy. However, some studies indicate significant improvement in practitioner efficiency. In fact, the grayscale DICOM GSDF was defined exactly for this purpose. Furthermore, appropriate measurement methods and the quality sensors for display characterization and calibration with accurate color calibrations of at least 11 bits for the internal display pipeline are beneficial. It is unclear what type of calibration is needed for medical applications, and whether each modality or specialty should have its own calibration method variant. Some modalities (e.g., dermatology, ophthalmology, and medical photography) would benefit from an absolute color calibration such that visualized images closely reflect reality, for instance, using color calibration charts commonly employed in professional photography and print [5]. However, there is no agreement concerning the type of calibration required for WSI, digital microscopy, histopathology, endoscopy, and laparoscopy. Absolute color calibration typically results in a reduction of color gamut, contrast, and luminance [23]. Therefore, absolute color calibration may not be the best alternative for these modalities. Perceptually, uniform calibration approaches resulting in color differences appearing equal in strength seem to be beneficial in all cases.

**Fig. 5:**
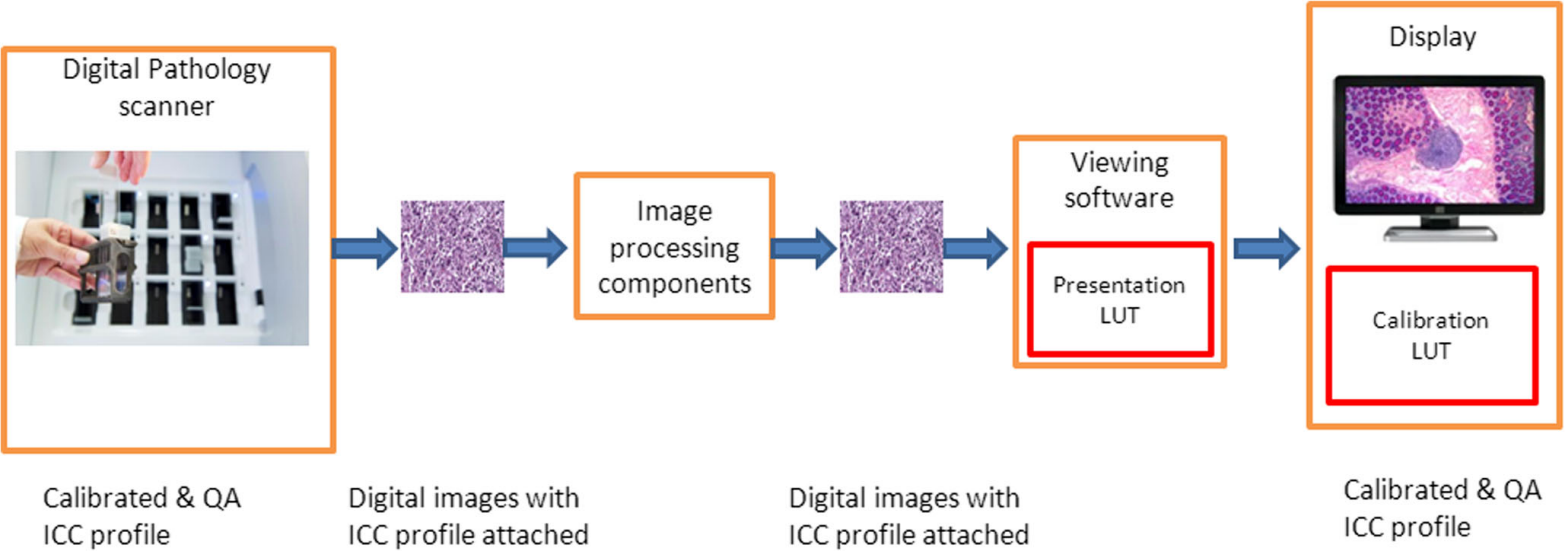
End-to-end calibrated visualization of the color information along the imaging chain

In addition, it has to be noted that display calibration is sensible only when the entire imaging chain is calibrated. In that context, architectures with a flexible end-to-end calibrated imaging chain are useful (see Fig. 5). For example, ICC profiles [24] describing the color space (range and metrics of the colorimetry of the device) and color behavior of the system or components could be used throughout the imaging chain. This would likely require defining a new rendering intent for medical content. At the display side, it would require defining a color extension of the widely accepted GSDF [22]. The display systems would need to be calibrated to reflect the newly defined color display function. However, the calibration of tablet and smart phone displays may prove to be particularly difficult, since most of these devices do not currently support ICC profiles.

## Telemedicine

Telemedicine uses digital images for remote diagnosis and management of medical conditions to reduce patient and clinician travel, improve access to subspecialist care, decrease wait times, and to reduce costs. Optimal acquisition and display of color medical images is critical to the diagnostic process as it impacts accuracy, discrimination, and consistency. Three aspects are relevant to color imaging in telemedicine as follows: acquisition, rendering, and display.

**Fig. 6:**
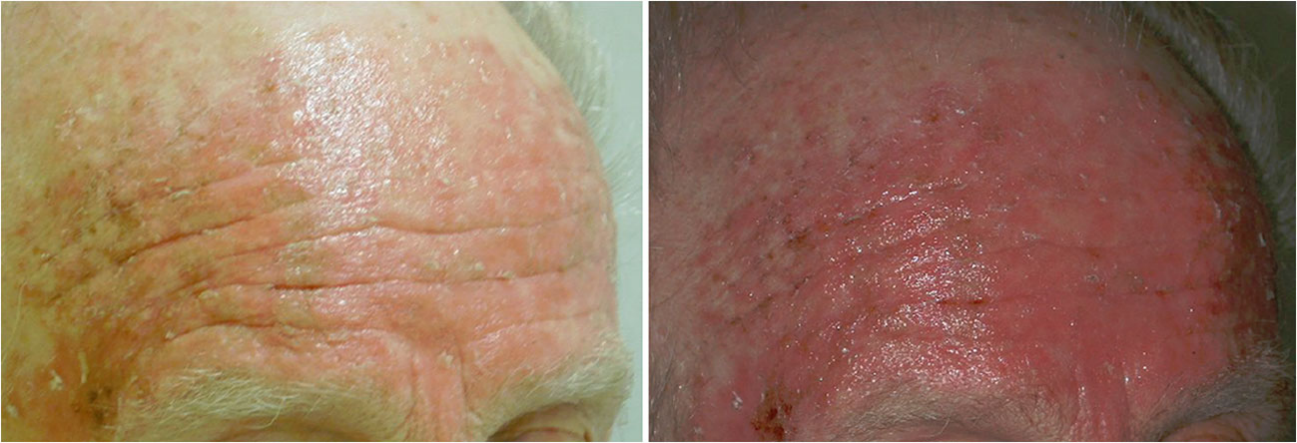
Example image data to be used to diagnose possible skin disease for the same patient and different hardware (image courtesy of Dr. Herbert Kirchesch)

**Fig. 7:**
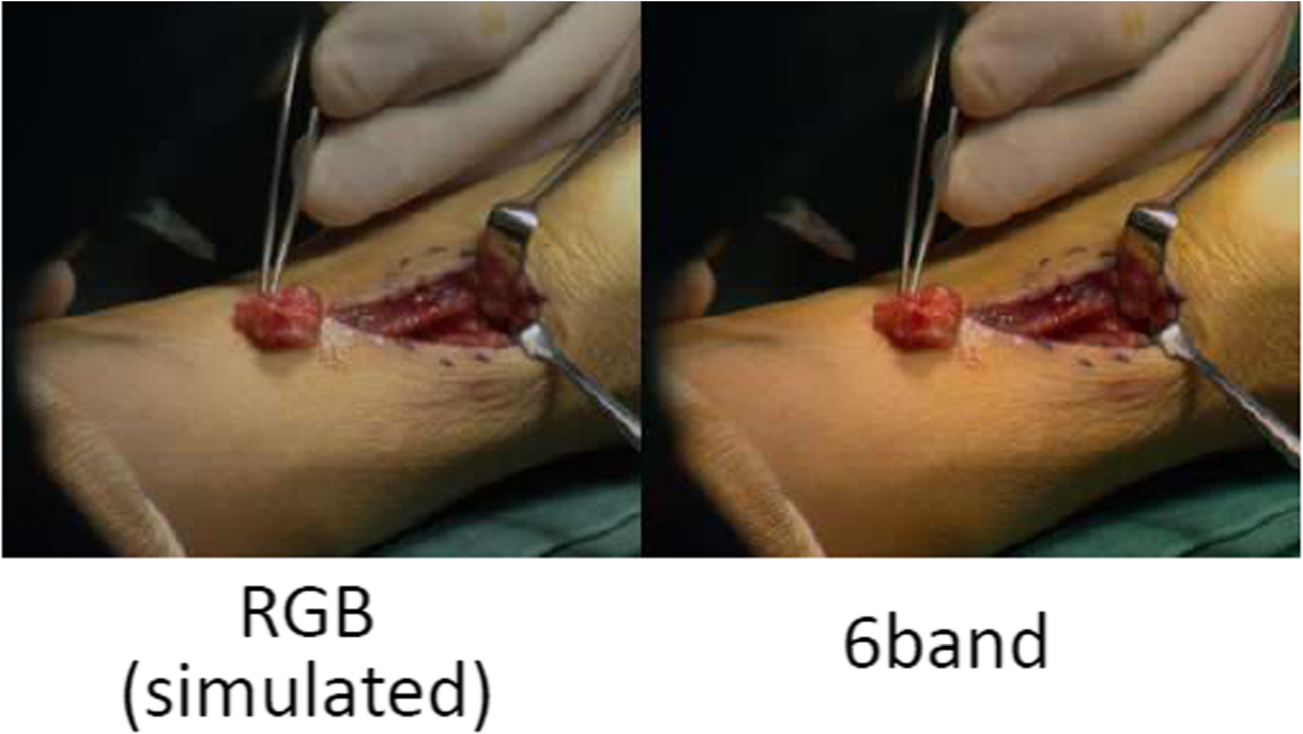
Observed color variation from RGB and six-band multispectral surgery image

Teledermatology (real-time and store-forward) shows why acquisition is important [25]. Providers send patient history and image data to a dermatologist who provides diagnostic and treatment recommendations, second opinions, monitoring, and/or e-learning, generally with a high degree of diagnostic accuracy (see Fig. 6). Conditions impacting color quality include lighting, camera settings (e.g., focus, flash, and white balance), compression, and views. Image rendering can improve quality, for example by using a spectrum-based reproduction system that produces high-fidelity color reproduction in different lighting environments. This type of system uses color or multispectral cameras (e.g., six-band video camera), devices for illumination spectrum measurements, calibrated color displays, and spectrum-based color conversion [26, 27]. Multispectral images from dermatology, surgery (see Fig. 7), and pathology have been rated by clinicians as achieving higher color reproducibility, better image fidelity, and superior appearance of material surface compared to conventional RGB-based images. Multispectral images also allow for quantitative color analyses that could improve diagnostic interpretations. For display, calibration and characterization protocols can be considered for high color accuracy. One pathology study [28] has revealed a slight advantage diagnostically for a properly calibrated and color-managed display and a significant advantage in terms of workflow.

Although teleclinicians emphasize the importance of accurate color in diagnoses and other clinical applications that use color images, there is a significant lack of data showing that poor or inappropriate calibration or standardization of acquisition and display devices can affect diagnostic performance and therefore there is no universally accepted image quality program for color displays. Although some technical telemedicine guidelines do exist, they rarely incorporate color calibration issues.

To optimize acquisition and display of color medical images, the consensus recommendations included standardization of data formats, exploration of automated color correction options, the development of methods and criteria for color reproduction capabilities of input and display devices, and performing studies to quantify the impact of color quality on a variety of clinically relevant diagnostic tasks from the perspectives of both the human observer and computer-based image analysis schemes.

To facilitate consistent and appropriate handling of color in telemedicine, the key recommendations included developing training methods for improving basic medical photography skills, incorporating standardized yet application-relevant color considerations into standards and guidelines documents, and increasing awareness of the importance of color quality.

## Standards and Recommendations

Standards are critical to color in medical imaging as they enable regulatory review and facilitate innovation. A well-specified implementation pathway with good quality, calibrated display systems supports greater diagnostic effectiveness and consistency. There is a complex web of standards-making bodies active in topics relating to color in medical imaging. Foremost among these are DICOM, American Association of Physicists in Medicine (AAPM), ICC, International Commission on Illumination (CIE), and International Engineering Consortium (IEC). DICOM specifies an extensible medical imaging file format that supports a variety of imaging modalities, can carry many different types of metadata, and is associated with an object-oriented network protocol and storage and retrieval services [29]. AAPM is responsible for standards in the areas such as radiology, cardiology, pathology, and dermatology. ICC defines the file format that carries color transforms (look-up tables) [17], and also documents the architectures and workflows for using the transforms. CIE is the international standards body for color and illumination, with interests in color, vision, lighting, and imaging applications. Other standards-making bodies active in this area include IEC, Video Electronics Standards Association (VESA), International Committee for Display Metrology (ICDM), and American College of Radiology (ACR). Although many of the necessary standards exist, they are not widely implemented in medical imaging. DICOM in particular supports advanced color capabilities through embedded ICC profiles, yet few if any actual products have the capability to write ICC profiles into the DICOM file, or to parse the profile and apply the transform correctly in the workflow. Another area where medical color applications would benefit from more rigorous application of standards and performance testing is in color displays. There is also some development work needed to define best practices for ambient lighting, multispectral imaging (imaging with an extended range and multitude of color sources), and archival color. A path toward improved standardization should include the implementation guidelines for each DICOM imaging modality and corresponding conformance tests, and the availability of source code for baseline implementations, possibly by connecting the ICC’s open source “Sample ICC” color management module (CMM) with a suitable open source DICOM viewer.

Beyond the need for implementation support of existing technologies, DICOM standards need to provide support for camera RAW image data to derive the colorimetry of a scene and for multispectral image data. In addition, other standards activities including AAPM and IEC work to evaluate color spaces and white points (due to report in 2014), and studies for objective comparison of colorimeters used in display calibration and profiling are in progress. These efforts will likely be useful if the standards bodies coordinate activities through liaisons in technical committees.

## Summary

It is clear from the number of attendees and level of participation in the color summit that experts from many areas of medical imaging realize the need for some degree of standardization of color in medical imaging. In some areas, color communication is to some extent successful based on the adoption (either deliberately or by default) of a sRGB model. In most of the identified areas, such a model is limited and a broader framework is needed. This broader framework should be able to provide support for sRGB images while at the same time providing for new technologies such as wide-gamut displays, scene capture, and multispectral imaging. The ICC model seems to be a good candidate and this was one of the reasons that led to the setting up of the ICC medical imaging working group to provide a focus for efforts to standardize color and to ensure that work in different medical imaging disciplines is well coordinated.

It is unrealistic to expect instant universal adoption of any proposed color framework. That said, for some modalities such as digital histopathology and ophthalmology, the lack of a well-defined color framework is limiting the use of digital tools that could maximize the utilization of medical devices for improved diagnostics. The recommendations in this paper are being implemented with the leadership of the recently created ICC medical imaging working group. The role of the ICC will be to provide a forum for discussion and implementation in close liaison with other medical imaging communities in order to ensure good adoption of well-defined color framework.

Moreover, user education in the area of color capture, processing and display, and future conferences with a focus on color should be considered for in-depth coverage of color issues for each imaging modality. In addition, the development of standard test methods for common functions and opportunities for manufacturers to test interoperability of systems can contribute further to a solid color framework across medical imaging applications with close collaboration with relevant standardization committees.

## Action Items and Work in Progress

In summary, a standard framework for color would provide several benefits for medical imaging applications. In some application areas, this benefit is expected to be substantial; whereas in other areas, the improvement is likely to be marginal. As a result of the discussions during the summit, the following candidate work items were identified.

- Calibration slide for histopathology. One reason for differences in whole slide imaging is the lack of a suitable calibration process which means that the same slide can look very different from system to system. Vendors that have worked in this area should pool their resources to develop a calibration system for digital microscopes.
- DICOM camera raw support and exchangeable image file format (Exif) tags. Today, DICOM provides support for JPEG images. However, the objective for these images is not usually colorimetric but to provide a good looking image. Work has begun to extend DICOM to provide support for Camera RAW image format and to include metadata from Exif tags to provide the data needed to derive the colorimetry of a scene.
- Medical RGB color space–mRGB. There is no suitable color display calibration objective for medical imaging displays designed to display color images. Work has begun to define a set of color spaces and ICC profiles for medical displays using the GSDF as the grayscale mRGB.
- Framework for multispectral imaging. Some medical imaging modalities are increasingly utilizing multispectral features but no suitable framework is defined for their storage, communication, and display. Proposal: define a multispectral imaging framework.
- Open source reference implementation. Integrating ICC profiles with viewer software can be difficult and sometimes results in a product with poor performance. Work has begun to connect the ICC’s open source “Sample ICC” or similar CMM with a suitable open source DICOM viewer to serve as a baseline implementation.
- Color support for mobile devices. Mobile or handheld display devices (e.g., smart phones and tablets) do not usually support ICC-based color management directly and any color framework needs to be able to accommodate this class of device. Guidelines for color support on mobile devices will benefit their use in the medical imaging field.
- Best practice papers for color in DICOM. The current DICOM framework could provide support for accurate color if used correctly but in many (most) cases color metadata (ICC profiles and other metadata) is ignored. Work has begun to develop guidelines for each imaging modality and where possible a set of tests that can be used to check conformance.
- Connectathon to check color capability. There is currently no way for system developers to check that color aspects of their latest developments are compatible with other products. We propose to encourage work around current connectathons that already provide an environment where developers can work directly with each other with technically competent arbiters present and these could be extended to include color aspects.
- Wiki for test images for all modalities. The lack of qualified test images often makes development of viewer software difficult. Having a set of reference images for all modalities may help to improve color presentation and a wiki could be set up to provide test images.
- Best practices for digital photography in medicine. There are many cases where color plays an important role in the diagnosis of disease from a medical image but it is not easy for a medical photographer to know how best to capture and communicate images. Work has begun to develop best practice guidelines for medical photography including jpeg and raw use cases.
- Calibration standard for ophthalmology. One reason for the color differences in the appearance of the retina in fundus imaging in ophthalmology is the lack of a suitable calibration method or standard. This causes significant retinal color disparity from camera to camera, even within the same manufacturer for the same patient. Work has begun to develop a suitable calibration phantom and calibration method, and devise the best working/vendor practices to ensure color consistency across devices and manufacturers.

## References

[1] Yagi Y: Color standardization and optimization in whole slide imaging. Diagn Pathol, 6(Suppl 1):S15, 2011. 10.1186/1746-1596-6-S1-S15 PMC3073208 21489185

[2] Hu E, Nosato H, Sakanashi H, Murakawa M (2013). A modified anomaly detection method for capsule endoscopy images using non-linear color conversion and higher-order local auto-correlation (HLAC). Conf Proc IEEE Eng Med Biol Soc.

[3] Glatz J, Varga J, Beatriz Garcia-Allende P, Koch M, Greten FR, Ntziachristos V (2013). Concurrent video-rate color and near-infrared fluorescence laparoscopy. J Biomed Opt.

[4] Narasimha-Iyer H, Can A, Roysam B, Stewart CV, Tanenbaum HL, Majerovics A, Singh H (2006). Robust detection and classification of longitudinal changes in color retinal fundus images for monitoring diabetic retinopathy. IEEE Trans Biomed Eng.

[5] Schalenbourg A, Zografos L (2013). Pitfalls in colour photography of choroidal tumours. Eye.

[6] Michalec GS (2006). The accuracy of digital retinal imaging (DRI) to screen for diabetic retinopathy: An analysis of two digital retinal imaging systems using standard stereoscopic seven-field photography and dilated clinical examination as reference standards. Journal of Ophthalmic Photography.

[7] Curtin RE (2007). Consistent color calibration for digital ophthalmic photography capture systems: principle and application. Journal of Ophthalmic Photography.

[8] Hubbard LD, Neider M, Thayer D, Wabers H, Vargo P, Lambert E (2007). Optimization and standardization of digital color fundus photographs and fluorescein angiograms: observations from a central reading center. Journal of Ophthalmic Photography.

[9] Madjarov B, Alexander J, Elsner K, Whitock R (2002). Design and implementation of standardized system for management of digital fundus images. Journal of Ophthalmic Photography.

[10] Nishibori M, Tsumura N, Miyake Y (2004). Why multispectral imaging in medicine?. Journal of Imaging Science and Technology.

[11] Korotkov K, Garcia R (2012). Computerized analysis of pigmented skin lesions: a review. Artif Intell Med.

[12] Penczek J, Boynton PA, Splett JD (2014). Color error in the digital camera image capture process. J of Digit Imag.

[13] Ahmad I (2009). Digital dental photography. part 5: lighting. Br Dent J.

[14] Bengel WM: Digital photography and the assessment of therapeutic results after bleaching procedures. J Esthet Restor Dent, 15 Suppl 1:S21–32; discussion S32, 2003 10.1111/j.1708-8240.2003.tb00315.x 15000901

[15] Bentley C, Leonard RH, Nelson CF, Bentley SA (1999). Quantitation of vital bleaching by computer analysis of photographic images. J Am Dent Assoc.

[16] Lasserre JF, Pop-Ciutrila IS, Colosi.HA: A comparison between a new visual method of colour matching by intraoral camera and conventional visual and spectrometric methods. J Dent, 39 Suppl 3:e29–e36, Dec 2011 10.1016/j.jdent.2011.11.002 22101123

[17] McLaren EA, Schoenbaum T: Combine conventional and digital methods to maximize shade matching. Compend Contin Educ Dent, 32 Spec No 4:30, 32–30, 33, 2011 22195347

[18] Moncada G, Silva F, Angel P, Oliveira O, Fresno M, Cisternas P, Fernandez E, Estay J, Martin J (2013). Evaluation of dental restorations: a comparative study between clinical and digital photographic assessments.

[19] Sluzker A, Knösel M, Athanasiou AE (2011). Sensitivity of digital dental photo CIE L*a*b* analysis compared to spectrophotometer clinical assessments over 6 months. Am J Dent.

[20] Torlakovic L, Olsen I, Petzold C, Tiainen H, Ogaard B (2012). Clinical color intensity of white spot lesions might be a better predictor of enamel demineralization depth than traditional clinical grading. Am J Orthod Dentofacial Orthop.

[21] Cheng WC, Keay T, O’Flaherty N, WangJ, Ivansky A, Gavrielides MA, Gallas BD, Badano A: Assessing color reproducibility of whole-slide imaging scanners. In SPIE Medical Imaging, pages 86760O–86760O. International Society for Optics and Photonics, 2013.

[22] Samei E, Badano A, Chakraborty D, Compton K, Cornelius C, Corrigan K, Flynn MJ, Hemminger B, Hangiandreou N, Johnson J (2005). Assessment of display performance for medical imaging systems: executive summary of AAPM TG18 report. Medical physics.

[23] Morovic J, Ronnier Luo M (2001). The fundamentals of gamut mapping: a survey. Journal of Imaging Science and Technology.

[24] ISO 15076–1. Image technology colour management—Architecture, profile format and data structure, 2010

[25] Bergmo TS, Wangberg SC, Schopf TR, Solvoll T (2009). Web-based consultations for parents of children with atopic dermatitis: results of a randomized controlled trial. Acta Paediatr.

[26] Yamaguchi M, Kishimoto J, Komiya Y, Kanno Y, Murakami Y, Hashizume H, Yamada R, Miyajima K, Haneishi H: Video-based telemedicine with reliable color: Field experiments of natural vision technology. In Proceedings of the 3rd International Universal Communication Symposium, pages 150–153. ACM, 2009

[27] Yamaguchi M, Murakami Y, Komiya Y, Kanno Y, Kishimoto J, Iwama R, Hashizume H, Aihara M, Furukawa M (2011). Video-telemedicine with reliable color based on multispectral technology.

[28] Krupinski EA, Silverstein LD, Hashmi SF, Graham AR, Weinstein RS, Roehrig H (2012). Observer performance using virtual pathology slides: impact of LCD color reproduction accuracy. Journal of Digital Imaging.

[29] ACR/NEMA. Digital Imaging and Communications in Medicine (DICOM), Part 3.14, Grayscale Standard Display Function, 2011. Technical report, January 2011

